# Oral Probiotics Ameliorate the Behavioral Deficits Induced by Chronic Mild Stress in Mice via the Gut Microbiota-Inflammation Axis

**DOI:** 10.3389/fnbeh.2018.00266

**Published:** 2018-11-06

**Authors:** Nannan Li, Qi Wang, Yan Wang, Anji Sun, Yiwei Lin, Ye Jin, Xiaobai Li

**Affiliations:** ^1^Department of Geriatrics Cardiology, First Hospital of China Medical University, Shenyang, China; ^2^Department of Psychiatry, The First Hospital of China Medical University, Shenyang, China; ^3^Department of Mental Health Center, China Medical University, Shenyang, China

**Keywords:** probiotics, *Lactobacillus*, *Bifidobacteria*, chronic mild stress, microbiota, inflammation, indoleamine 2, 3-dioxygenase

## Abstract

In recent years, a burgeoning body of research has revealed links between depression and the gut microbiota, leading to the therapeutic use of probiotics for stress-related disorders. In this study, we explored the potential antidepressant efficacy of a multi-strain probiotics treatment (*Lactobacillus helveticus R0052*, *Lactobacillus plantarum R1012*, and *Bifidobacterium longum R0175*) in a chronic mild stress (CMS) mouse model of depression and determined its probable mechanism of action. Our findings revealed that mice subjected to CMS exhibited anxiety- and depressive-like behaviors in the sucrose preference test, elevated plus maze, and forced swim test, along with increased interferon-γ, tumor necrosis factor-α, and indoleamine 2,3-dioxygenase-1 levels in the hippocampus. Moreover, the microbiota distinctly changed from the non-stress group and was characterized by highly diverse bacterial communities associated with significant reductions in *Lactobacillus* species. Probiotics attenuated CMS-induced anxiety- and depressive-like behaviors, significantly increased *Lactobacillus* abundance, and reversed the CMS-induced immune changes in the hippocampus. Thus, the possible mechanism involved in the antidepressant-like activity of probiotics is correlated with *Lactobacillus* species via the gut microbiota-inflammation-brain axis.

## Introduction

Major depressive disorder (MDD) is a widespread debilitating neuropsychiatric disorder that is the leading cause of early mortality from suicide and results in huge socioeconomic impacts (Hasin et al., 2018); however, its pathogenesis has not been sufficiently elucidated. Inadequate treatment of depression and poor treatment efficacy (only 30% of MDD cases are in remission after first-line antidepressant therapies) are worldwide issues (Valdes-Tovar et al., 2018). Thus, recent efforts have focused on developing better treatment approaches for MDD.

Emerging evidence from animal models and human patients over the past decade have highlighted the potential contribution of the gut microbiota in MDD pathogenesis (Galley et al., 2014; Naseribafrouei et al., 2014; Gacias et al., 2016; Zheng et al., 2016; Huo et al., 2017; Marin et al., 2017); however, the characteristic changes of the gut microbiota in animal models and depression patients have been disparate (Gacias et al., 2016; Zheng et al., 2016; Bridgewater et al., 2017). The gut microbiota is a core component of the brain-gut axis that can alter behaviors through neurotransmitters and neuropeptides (O’Mahony et al., 2015; Reigstad et al., 2015), low grade immune activation (de Punder and Pruimboom, 2015), hypothalamic-pituitary-adrenal axis activity (Huo et al., 2017), and neurogenesis (Mohle et al., 2016). Despite these clear links, the precise mechanisms of the brain-gut axis remain poorly understood. Recent animal and human studies have suggested that gut microbiota interventions including probiotics, antibiotics, dietary supplement, or fecal microbiota transplantation may be effective treatments for neuropsychiatric disorders (Gacias et al., 2016; Wang et al., 2016; Kazemi et al., 2018; Vermeulen et al., 2018). The beneficial effects of probiotics in alleviating psychological disorders are associated with protecting the intestinal barrier, improving mucosal and systemic immunity, normalizing the exaggerated hypothalamic-pituitary-adrenal axis, and restoring neurotransmitters and brain-derived neurotrophic factor levels (Galley et al., 2014; de Punder and Pruimboom, 2015; Liang et al., 2015; Dhaliwal et al., 2018). Probiotics are most frequently of the *Lactobacillus* or *Bifidobacterium* species. Most studies have reported that *Lactobacillus* levels decreased after stress (Galley et al., 2014; Marin et al., 2017; Dhaliwal et al., 2018), and that administering a single strain of *Lactobacillus* was sufficient to improve stress-induced behaviors (Liang et al., 2015; Bharwani et al., 2017; Dhaliwal et al., 2018). Additionally, some studies have found that *Bifidobacteria* also had positive effects on stress-related diseases (Savignac et al., 2014; Pinto-Sanchez et al., 2017; Meyer and Vassar, 2018); however, the exact mechanisms of these effects remain unclear.

Inflammation has long been recognized as a causative factor in psychological stress and psychiatric disorders including MDD (Lichtblau et al., 2013). The chronic production of inflammatory cytokines such as tumor necrosis factor-α (TNF-α), interferon (IFN)-γ, interleukin (IL)-1β, and IL-6 is closely related to the etiology of MDD (Laugeray et al., 2016; Noto et al., 2016; Pfau et al., 2018). Although, each inflammatory disease has its own unique particularities in pathogenesis, several studies have revealed that the gut microbiota contributes to the development of many complex inflammatory diseases (Consonni et al., 2018). The gut microbiota is also associated with systemic host immunity at both the steady state and inflammatory conditions (Consonni et al., 2018). In most tissues, optimal commensal bacteria can balance between stimulatory and regulatory signals, establish a threshold of activation and regulation that is required for immune fitness, and maintain tolerance to innocuous antigens (Belkaid and Hand, 2014). On the contrary, microbial disturbances that effect the immune system contribute to the onset of pathology and the rapid increase in chronic inflammatory and autoimmune disorders (Belkaid and Hand, 2014). Probiotics have been shown to have immunomodulatory functions through the systemic expansion of regulatory T cells, a population that highly expresses the anti-inflammatory cytokine IL-10 (Forsythe et al., 2007; Konieczna et al., 2012; Bharwani et al., 2017; Consonni et al., 2018).

Tryptophan (TRP) is an essential amino acid for the biosynthesis of proteins and the neurotransmitter serotonin. Serotonin is a monoamine that modulates central and peripheral functions and has been linked to the development of depression (Lichtblau et al., 2013). The probiotic strains *Lactobacillus* and *Bifidobacteria* have been reported to affect TRP metabolism (Liu et al., 2018). Indoleamine 2,3-dioxygenase-1 (IDO1) is the first rate-limiting intracellular enzyme in the catabolism of TRP to kynurenine (KYN) outside the liver. IDO1 is an immune mediator that is responsive to several proinflammatory mediators, including IFNs (IFN-α, -β, and -γ), TNF-α, and IL-6 (Murakami and Saito, 2013). Increased IDO1 activity has been found to be positively correlated with the severity of depressive scores, and could predict future risk for developing stress-related symptoms (Murakami and Saito, 2013; Wang et al., 2018), which due to systemic immune activation, drives TRP down the IDO1-mediated KYN pathway. Previous studies have found potential antidepressant efficacy by blocking IDO1 activity both in chronic stress- and inflammation-induced behavior models (Laugeray et al., 2016). Interestingly, IDO1 activity is correlated with microbial translocation and disrupted mucosal immunity (Chen et al., 2015). Recent research has found that gut-resident *Lactobacillus* depletion is associated with enhanced IDO1 activity (Vujkovic-Cvijin et al., 2015; Marin et al., 2017); however, in some inflammatory diseases, IDO1 expression may increase after supplementing several *Lactobacillus* strains (Forsythe et al., 2007; Tanoue et al., 2016).

Considering the extensive literature linking *Lactobacillus* and *Bifidobacteria* with stress-induced complications, we investigated a multi-strain probiotics treatment consisting of *Lactobacillus helveticus R0052, Lactobacillus plantarum R1012, and Bifidobacterium longum R0175* for its antidepressant potential in a chronic mild stress (CMS)-induced mouse model of depression. We further investigated the impact of behavior, gut microbiota changes, IDO1 expression, and inflammatory cytokines in the hippocampus to determine its possible mechanisms of action.

## Materials and Methods

### Animals

Six to eight weeks old male C57BL/6 mice were purchased from Beijing Vital River Laboratory Animal Technology. They were housed in groups (3–4) in plastic cages (290 mm × 178 mm × 150 mm, M1) under standard conditions (21 ± 1°C and 55 ± 2% humidity) on a 12 h light/dark cycle, with lights on at 7:30 AM, and had *ad libitum* access to food and water. Animals were adapted to the laboratory conditions for 2 weeks before the onset of the experiment. This study was carried out in accordance with the Guide for the Care and Use of Laboratory Animals published by the US National Institutes of Health and approved by the China Medical University Animal Care and Use Committee.

### Experimental Design

**FIGURE 1:**
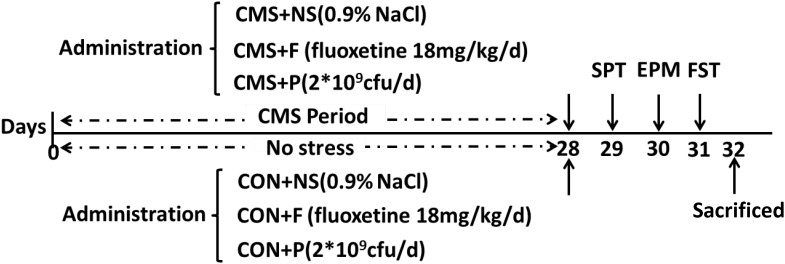
Experimental design. Representation of the study design. C57BL/6 mice were fed for a total of 4 weeks with a mixture of three probiotics (*Lactobacillus helveticus R0052, L. plantarum R1012*, *and Bifidobacterium longum R0175*), the antidepressant fluoxetine, or saline. After 4-week treatment, animals underwent a battery of testing relevant to anxiety. All groups were sacrificed on the same day. Cecal contents and the entire hippocampus tissue were harvested for further analysis. SPT, sucrose preference test; EPM, elevated plus maze; FST, forced swim test; CMS + NS, subjected to the CMS procedure and received saline; CMS + F, subjected to the CMS procedure and received fluoxetine; CMS + P, subjected to the CMS procedure and received probiotics; CON + NS, not subjected to any stress and received saline; CON + F, not subjected to any stress and received fluoxetine; and CON + P, not subjected to any stress and received probiotics.

The mice were randomly divided into six groups (CMS + NS: subjected to the CMS procedure and received saline, CMS + F: subjected to the CMS procedure and received fluoxetine, CMS + P: subjected to the CMS procedure and received probiotics, CON + NS: not subjected to any stress and received saline, CON + F: not subjected to any stress and received fluoxetine, and CON + P: not subjected to any stress and received probiotics). Each experimental group consisted of eight mice. Mice were fed daily with probiotics, fluoxetine, or saline in the corresponding groups using sterile gavage needles. All CMS groups were subjected to stress at the same time for 28 days (4 weeks) and all CON groups were uninterrupted except for feeding and cage cleaning. After 28 days, all mice underwent three consecutive days of behavioral testing (Figure 1).

### Chronic Mild Stress Protocol

The CMS paradigm was performed as previously described (Yang et al., 2017; Nikolova et al., 2018), which consisted of the following stressors: cage tilted at 45°(24 h), food and water deprivation (24 h), tail pinch (60 s, 1 cm from the end of the tail), reverse day and night (24 h), overnight stroboscopic lighting (400 flash/min in the dark, 10 h), restraint in a 50-ml plastic syringe tube (2 h), and moist bedding (200 mL of water spilled onto bedding, 8 h) for a total of 4 weeks. The same stressor was not applied on two consecutive days.

### Drugs

The probiotics used were a mixture of three strains (*L. helveticus R0052, L. plantarum R1012*, *and B. longum R0175*) as freeze-dried stocks (Lallemand Health Solutions, Mirabel, QC, Canada) that were reconstituted daily in saline solution to a final concentration of 10 × 10^9^ CFU/mL. The bacterial solution (200 μL or 2 × 10^9^ CFU) was administered by oral gavage daily for 4 weeks during the experimental procedure. CMS + NS and CON + NS groups were gavaged with equivalent volumes (200 μL) of saline solution. The selective serotonin reuptake inhibitor fluoxetine (Eli Lilly, Indianapolis, IN, United States) was used as a control antidepressant. Fluoxetine was made up fresh daily in saline solution, and the dose administered was 18 mg/kg by daily oral gavage (Dulawa et al., 2004).

### Behavioral Testing

#### Sucrose Preference Test (SPT)

Sucrose preference test is used as an indicator of anhedonia, a core symptom of depression. The SPT was conducted twice: first, at baseline prior to the start of the CMS protocol, also served as the training test, and second, after 4 weeks of CMS (On day 29) as part of a battery of behavioral tests. All mice were singly housed and presented with two preweighed bottles containing 1% sucrose solution or tap water. The position of the bottles was switched every 12 h to prevent the effects of side preference on drinking behavior. Water and sucrose solution intake was measured 24 h after introducing the sucrose solution by weighing the bottles. Sucrose preference was calculated as a percentage of the volume of sucrose consumed over the total volume of liquid consumed. After SPT all mice were place back into their home cages.

#### Elevated Plus Maze (EPM)

On day 30, EPM was performed to measure anxiety-related behaviors. EPM consisted of two opposite open arms (50 cm × 10 cm) and two opposite closed arms (50 cm × 10 cm) with 30 cm opaque high walls, and was elevated 50 cm above the ground. Each mouse was gently placed in the center of the maze and left to explore for 5 min. The frequency of the open and closed arms entries and the time spent in the open and closed arms were recorded using EthoVision. After each run, the arena was cleaned with 70% ethanol to eliminate any odor cues.

#### Forced Swim Test (FST)

Forced swim test is a well-characterized behavioral paradigm to assess depressive-like behavior in rodents. On day 31, mice were subjected to the FST. Mice were placed into glass cylinders (12 cm diameter, 23 cm deep) filled with 23–25°C water for 6 min. Swimming behaviors were recorded by a video camera. The video images were later analyzed using the video-tracking program EthoVision, version 7.0 (Noldus, Wageningen, Netherlands). The duration of immobility during the last 4 min of the test was quantified.

### Sample Collection

One day after the completion of behavioral tests, all mice were anesthetized (400 mg/kg chloral hydrate intraperitoneally), and subsequently sacrificed by decapitation. The brain was rapidly removed and placed on ice-cold plates, then the whole hippocampus was quickly dissected and frozen in liquid nitrogen immediately (Harkin et al., 2001). Right hippocampus sample was used for cytokine measurements, and left hippocampus was used for Western blotting. Simultaneously the cecal contents were collected and used for 16S rDNA gene sequence analysis. All the samples were stored at -80°C for further analysis.

### Hippocampal Cytokine Measurements

The concentration of cytokines (IFN-γ, TNF-α, IL-6, and IL-10) in the hippocampus was quantified using enzyme-linked immunosorbent assay kits (EK2802/2, EK2822/2, EK2062/2, and EK2102/2) from MultiSciences (Lianke Biotech, Hangzhou, China) according to the manufacturer’s specifications.

### Western Blotting

Hippocampi were prepared for western blotting, and protein concentration determined using BCA Protein Assay Kit (Thermo Fisher Scientific, Grand Island, NY, United States). Proteins were separated using sodium dodecyl sulfate-polyacrylamide gel electrophoresis, electrophoretically transferred to a polyvinylidene fluoride membrane, and incubated overnight with diluted antibody: rabbit polyclonal to IDO (45 KDa) (#106134; Abcam, Cambridge, United Kingdom; 1:50) at 4°C which does not cross react with IDO2 (Wainwright et al., 2014). This antibody was raised against a mixture of three rat IDO peptides: MPHSQISPAEGSRRILEEY (1–19), LFSFPGGDCDKGFFLVSLMVE (155–176), and VKPSKQKPMGGHKSEEPS (359–376). They were further incubated with the appropriate horseradish peroxidase-linked secondary antibody (biotinylated goat anti-rabbit; Boster Biological Technology, Wuhan, China), and immune complexes were detected with enhanced chemiluminescence (Santa Cruz Biotechnology, CA, United States). Finally, the quantitation analysis was performed using ImageJ software and normalized to the loading control protein β-actin.

### Fecal Microbiota Analysis

16S rDNA high-throughput sequencing were performed by Realbio Genomics Institute (Shanghai, China) using Illumina HiSeq PE250. Genomic DNA was extracted and then amplified targeting the V3-4 region with indexes and adaptor-linked universal primers (341F: ACTCCTACGGGAGGCAGCAG, 806R: GGACTACHVGGGTWTCTA AT). The raw paired-end reads were assembled and quality-filtered using PANDAseq (Masella et al., 2012). The qualified reads were clustered into generate operational taxonomic units (OTUs) at the 97% similarity level using USEARCH with QIIME software. OTU hierarchical clustering, species annotation, principal component analysis (PCA), alpha-diversity analysis, beta-diversity analysis, and species abundance analysis were performed using R.

### Statistical Analysis

All statistical analyses were performed using SPSS version 17.0 (SPSS Inc., Chicago, IL, United States). Data were analyzed using two-way (stress level ^∗^ drug administration) analysis of variance (ANOVA) followed by the Bonferroni *post hoc* test. Graphs were generated using GraphPad Prism 5 (GraphPad Software, La Jolla, CA, United States). For 16S rRNA gene sequencing data, the analysis and visualization of microbiome communities were performed using the R programming language and the Vegan package. β-diversity was determined by Anosim using both unweighted and weighted UniFrac metrics. Visualization of all sequenced samples was performed using principle coordinate analysis (PCoA). The significance of relative abundances of microbial compositions and the α-diversity analysis were determined using the Kruskal-Wallis (>2 groups) or Wilcoxon rank sum (2 groups) tests. For Kruskal-Wallis tests, if *P*-values were significant (*P* < 0.05), then pairwise differences were calculated. Statistical significance was assessed using R 3.0.2. A value of *P* < 0.05 was considered statistically significant. No animals or data points were excluded from analyses.

## Results

### Probiotics Ameliorated CMS-Induced Anxiety- and Depressive-Like Behaviors

**FIGURE 2:**
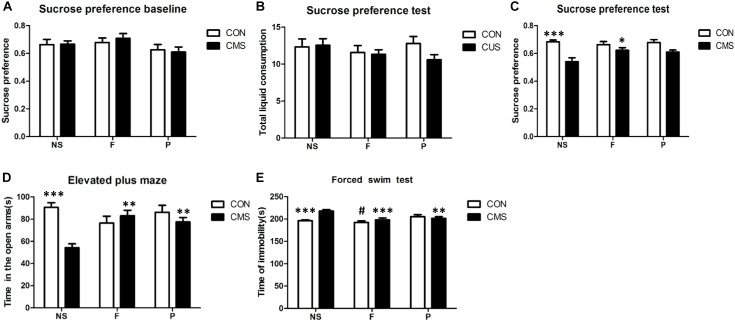
Behavioral assessments in mice. **(A)** There were no differences among six groups in sucrose preference at baseline before exposure to the CUS protocol. **(B)** SPT results showed no significant differences in total liquid consumption. **(C)** Sucrose preference was significantly decreased after 4 weeks of CMS, and this effect of stress was reversed by fluoxetine, but not by probiotics. **(D)** EPM test results showed that the time spent in the open arms was significantly reduced in the CMS group, and this effect of stress was reversed by fluoxetine and probiotics. **(E)** Results of FST showed that the immobility time was significantly increased in the CMS group; the change of immobility time induced by CMS was reversed by fluoxetine administration or probiotics administration. The comparision of three groups without CMS revealed that fluoxetine administration led a significantly decreased in the immobility time compared with probiotics administration. Data are presented as mean ± SEM; *n* = 8 per group; ^∗^*P* < 0.05, ^∗∗^*P* < 0.01, ^∗∗∗^*P* < 0.001 vs. the CMS + NS group, ^#^*P* < 0.05 vs. the CON + P group. SPT, sucrose preference test; EPM, elevated plus maze; FST, forced swim test; CMS + NS, subjected to the CMS procedure and received saline; CMS + F, subjected to the CMS procedure and received fluoxetine; CMS + P, subjected to the CMS procedure and received probiotics; CON + NS, not subjected to any stress and received saline; CON + F, not subjected to any stress and received fluoxetine; and CON + P, not subjected to any stress and received probiotics.

In the baseline phase, the sucrose preference index did not differ significantly among the six groups [*F*(2, 42) = 0.234, *P* = 0.792]. The CMS protocol effectively induced anxiety- and depressive-like behaviors in SPT, EPM, and FST. All behavioral changes induced by the CMS protocol were reversed by fluoxetine administration. Probiotics treatment partially reversed the anxiety-like behavior in EPM and despair behavior in FST, however, no significant increased preference for sucrose was found in SPT (Figure 2).

As measured by SPT, total liquid intake did not significantly differ among the groups without main and interaction effects, however, the 1% sucrose solution consumption revealed a significant main effect of stress level [*F*(1,42) = 25.402, *P* < 0.001] and interaction effect of stress level and drug administration [*F*(2,42) = 3.340, *P* = 0.045], while no significant effect of drug administration was found [*F*(2,42) = 1.592, *P* = 0.215]. *Post hoc* tests found that sucrose preference was decreased significantly by CMS (CON + NS vs. CMS + NS, *P* < 0.001), and the effect of CMS could be reversed by fluoxetine administration (CMS + F vs. CMS + NS, *P* = 0.021). In the EMP test, two-way ANOVA found a significant interaction of stress level and drug administration [*F*(2,42) = 9.672, *P* < 0.001] of the time spent in open arms, and stress level had main effects [*F*(1,42) = 10.069, *P* = 0.003] but not drug administration [*F*(2,42) = 2.006, *P* = 0.147]. A significant decrease of time spent in open arms was found in CMS + NS mice compared with CON + NS mice (*P* < 0.001), CMS treated with fluoxetine or probiotics showed increased time spent in open arms compared with the CMS + NS group (*P* = 0.001 and *P* = 0.006, respectively). The FST immobile time differed significantly with interaction effects of stress level and drug administration [*F*(2,42) = 7.699, *P* = 0.001] among groups, both stress level [*F*(1,42) = 4.21, *P* = 0.046] and drug administration [*F*(2,42) = 9.833, *P* < 0.001] had main effects on the immobile time. *Post hoc* analysis revealed that CMS + NS group mice were more the immobile than the CON + NS (*P* < 0.001), and this effect was reversed by fluoxetine administration (CMS + F vs. CMS + NS, *P* < 0.001) or probiotics administration (CMS + P vs. CMS + NS, *P =* 0.006). In addition, *post hoc* analysis of the three groups without any stress revealed that fluoxetine administration significantly reduced the immobile time compared with probiotics administration (CON + F vs. CON + P, *P* = 0.033). Fluoxetine and probiotics improved the despair behavior induced by CMS (Figure 2).

### Differences in Microbial Community Structure Between Groups

**FIGURE 3:**
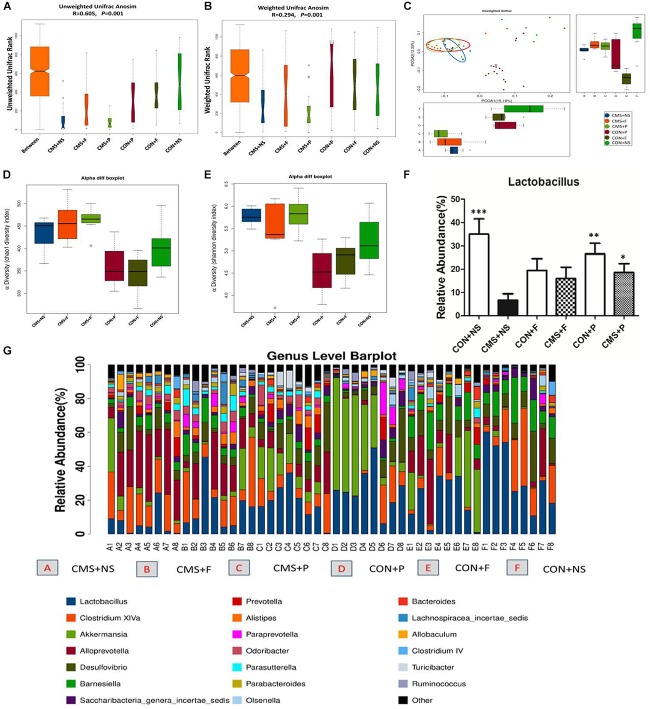
Alterations of gut microbiota. **(A)** Unweighted UniFrac Anosim was significantly different among groups. **(B)** Weighted UniFrac Anosim was significantly different among groups. **(C)** Two-dimensional principal coordinate analysis (PCoA) of unweighted UniFrac distances showed a clear difference in the microbial community compositions between the stress and non-stress groups. **(D)** The α-diversity index, Chao1 revealed differences in species richness; stress-exposed samples presented significantly higher diversity. **(E)** The α-diversity index, Simpson revealed differences in species evenness; stress-exposed samples presented significantly higher species evenness. **(F)** Relative abundance of the genus *Lactobacillus* in the six groups, ^∗^*P* < 0.05, ^∗∗^*P* < 0.01, ^∗∗∗^*P* < 0.001 vs. the CMS + NS group. **(G)** The most abundant taxa at the genus level in all samples; *n* = 8 per group.

β-diversity analyses of Anosim based on unweighted UniFrac ranks and weighted UniFrac distance ranks, which focuses on the degree of microbial phylogenetic similarity, displayed that vastly different bacterial community structures existed between the six groups (*R* = 0.605, *P* = 0.001 and *R* = 0.294, *P* = 0.001, respectively) (Figures 3A,B). Visualization of all sequenced samples via PCoA displayed site-specific clustering. PCoA based on unweighted UniFrac revealed obvious clustering of the CMS + NS, CMS + F, and CMS + P groups (Figure 3C). The microbiota within the stressor-exposed samples were significantly different than those from the non-stress groups, which might indicate exposure to stress disrupted existing intestinal microbiota profiles.

### Differences in Microbial Richness and Evenness Between Groups

The α-diversity determines the richness and evenness within bacterial populations. The α-diversity analysis showed that there were significant differences among the six groups. The α-diversity index, Chao1 (*P* < 0.0001), revealed differences in species richness, and the Simpson index (*P* = 0.0001), revealed differences in species evenness. We found higher diversity and species evenness in stress-exposed samples, but there were no significant differences between the CMS + NS, CMS + F, and CMS + P groups (Figures 3D,E).

### Differences in Microbial Composition

The intestinal microbiota of all samples mainly belonged to only two phyla, *Firmicutes* and *Bacteroidetes* (86.7 ± 9.1%). We next applied the non-parametric Kruskal-Wallis test to the genus-level microbiota data to determine if exposure to the stressor significantly changed the relative abundance of *Lactobacillus* (*P* = 0.025). Stress depleted the *Lactobacillus* compartment. The CMS + NS mice had a significantly decreased proportion of *Lactobacillus* (6.6 ± 2.8%) compared with the CON + NS (35.1 ± 18.1%, *P* < 0.001) and CON + P (26.6 ± 13.0%, *P* = 0.003) groups. CMS + P mice (18.6 ± 10.5%) had a higher abundance of *Lactobacillus* compared with CMS + NS (*P* = 0.028) mice; however, there were no significant differences in the mice that received fluoxetine [CON + F (19.5 ± 14.1%, *P* = 0.05) and CMS + F (16.0 ± 13.7%, *P* = 0.083)] (Figures 3F,G).

### Differences in Inflammatory Cytokine Levels in the Hippocampus Differed Significantly

**FIGURE 4:**
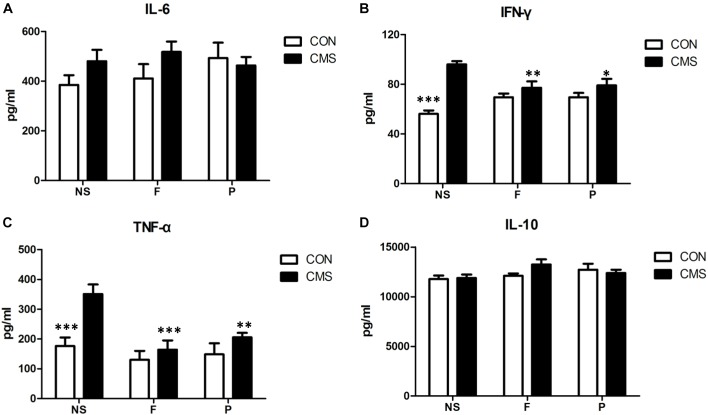
Cytokine concentrations in the hippocampus. **(A)** There was no significant difference in hippocampal IL-6 level among six groups. **(B)** CMS significantly increased IFN-γ expression in the hippocampus, which could be reversed by supplementation with probiotics or fluoxetine. **(C)** CMS significantly increased TNF-α level in the hippocampus, which could be reversed by supplementation with probiotics or fluoxetine. **(D)** There was no significant difference in hippocampal IL-10 level among six groups. Data are presented as mean ± SEM; *n* = 8 per group; ^∗^*P* < 0.05, ^∗∗^*P* < 0.01, ^∗∗∗^*P* < 0.001 vs. the CMS + NS group.

The proinflammatory cytokines IFN-γ, IL-6, and TNF-α and the anti-inflammatory cytokine IL-10 were detected in the hippocampus to evaluate inflammatory status. Two-way ANOVA results for the interaction and main effects of hippocampal IL-6 and IL-10 levels were non-significant. IFN-γ expression revealed a significant interaction effect of stress level and drug administration [*F*(2,42) = 11.056, *P* < 0.001] and main effect of stress level [*F*(1,42) = 36.967, *P* < 0.001]. *Post hoc* tests found that there was a significant rise in hippocampal IFN-γ expression by CMS (CON + NS vs. CMS + NS, *P* < 0.001), and CMS with supplementation of fluoxetine or probiotics could reduced hippocampal IFN-γ level (CMS + F vs. CMS + NS, *P* = 0.004 and CMS + P vs. CMS + NS, *P* = 0.01). Both stress level [*F*(1,42) = 13.394, *P* = 0.001] and drug administration [*F*(2,42) = 8.343, *P* = 0.001] had significant effects on the hippocampal TNF-α expression, but no statistical differences of interaction effect were found. Additionally, a significant increase of hippocampal TNF-α expression was found in CMS + NS mice compared with CON + NS mice (*P* < 0.001), CMS with fluoxetine or probiotics supplementation was associated with lower hippocampal TNF-α level (CMS + F vs. CMS + NS, *P* < 0.001 and CMS + P vs. CMS + NS, *P* = 0.004) (Figure 4).

### Differences in IDO1 Protein Levels in the Hippocampus

**FIGURE 5:**
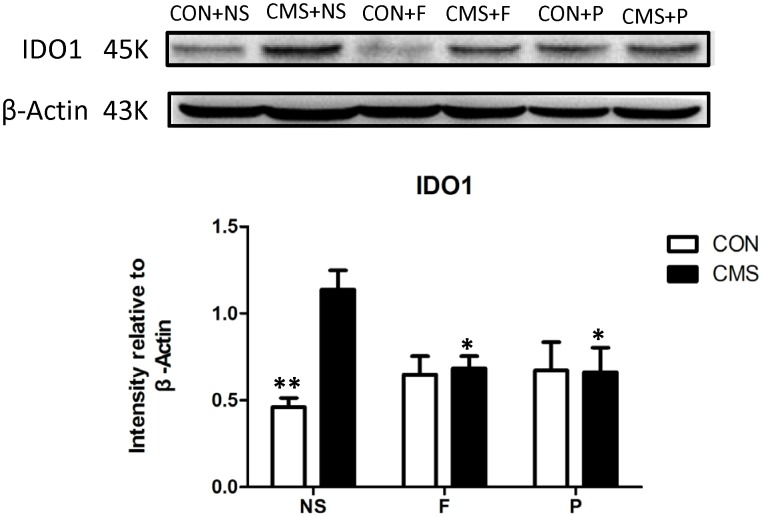
IDO1 protein levels in the hippocampus. Above: western blots for IDO1. Below: ImageJ analysis of IDO1 protein intensity normalized to β-actin; *n* = 4 per group; ^∗^*P* < 0.05; ^∗∗^*P* < 0.01.

Two-way ANOVA showed that hippocampal IDO1 expression differed significantly with interaction effects of stress level and drug administration [*F*(2,18) = 5.653, *P* = 0.012] among groups, stress level [*F*(1,18) = 6.355, *P* = 0.021] had main effect. *Post hoc* testing showed that CMS + NS group mice had higher IDO1 protein levels in the hippocampus than the CON + NS (*P* = 0.001). Administering probiotics and fluoxetine also significantly reduced IDO1 protein levels in the hippocampus (CMS + NS vs. CMS + F, *P* = 0.035 and CMS + P vs. CMS + NS, *P* = 0.026) (Figure 5).

## Discussion

In this study, we provide evidence that the mixture of three probiotic strains (*L. helveticus R0052, L. plantarum R1012*, and *B. longum R0175*) could alleviate CMS-induced anxiety- and depressive-like behaviors in mice. In addition to behavioral changes, we also found neuro-inflammation and gut microbiota changes in mice. CMS is a well-established animal model of depression that has been shown to result in brain-gut axis dysfunctions (Burokas et al., 2017). In our study, after 4-week CMS exposure, SPT, EPM, and FST demonstrated that the tested animals had acquired anxiety- and depressive-like behaviors. Furthermore, fluoxetine completely ameliorated these CMS-induced phenotypes, while probiotics partially improved EPM and FST following CMS exposure. Probiotics did not effectively improve symptom of anhedonic behavior in SPT of CMS mice, however, normalization of FST behaviors has proven to be a reliable marker of antidepressant activity (Cryan et al., 2005). In stress-free mice, fluoxetine slightly reduced the immobile time in FST, but probiotics did not. Probiotics showed modest antidepressant effects compared with fluoxetine. The effect of probiotic supplementation on neuro-behavioral and mood outcomes in this study are consistent with previous studies (Wang et al., 2016). We next demonstrated the effect of CMS on the functional profiles and composition of the gut microbiota: CMS resulted in increased species diversity and richness and significant community profile divergence from the non-stress groups. The α-diversity indices Chao1 and Simpson showed that CMS increased the richness and evenness within bacterial populations, similar to some previous results (Naseribafrouei et al., 2014; Jiang et al., 2015), but in contrast to others (Bharwani et al., 2017). Importantly, we found that stress primarily depleted *Lactobacillus* abundance, while administering the probiotic strains *L. helveticus R0052, L. plantarum R1012*, and *B. longum R0175* reversed such changes in microbiota composition. These behavioral and gut microbiota aberrations were paralleled by biochemical alterations including higher levels of IDO1 and proinflammatory cytokines in the hippocampus. Our observations suggested that gut microbiota disturbances were associated with the pathophysiology of depression (Bruce-Keller et al., 2018). The beneficial behavioral effects of probiotics might involve downregulating activated IDO1 via inhibiting neural pro-inflammatory cytokine activities.

Major depressive disorder is associated with profound changes in the composition of the intestinal microbiota, highlighting the importance of the microbiota in disease etiology. While the precise roles of probiotics in depressive states and other stress-related disorders remain under-explored, this study suggested that probiotics exerted neuro-behavioral effects by restoring the microbiota, which influenced the effects of chronic stress on host immunity. A recent study showed that CMS caused damage to gastric mucosa, intestinal microvilli, and increased inflammatory cell invasion, which can be gradually restored to normal following Bifidobacterium or fluoxetine treatment (Zhu et al., 2017). We found that higher diversity and evenness within cecum bacterial populations was associated with reduced *Lactobacillus* in the CMS + NS group; therefore, it is reasonable to presume that highly diverse bacterial communities associated with reduced *Lactobacillus* abundance are likely to be involved in the pathogenesis of stress-induced behavioral alterations. Zevin et al. (2016) showed that low diversity communities dominated by *Lactobacillus* species were associated with maintaining female genital tract health via protecting epithelial barriers and inhibiting inflammation. We found that cecal microbiota analyses of the non-stress with probiotics treatment group showed low diversity communities dominated by *Lactobacillus* species. CMS following probiotics or fluoxetine treatment also showed high diversity bacterial communities similar to the CMS + NS group, whereas the associated increase in *Lactobacillus* abundance could partially reverse stress-induced emotional alterations. Our results indicated that alterations in *Lactobacillus* may be associated with depressive- and anxiety-like behaviors in mice.

Indoleamine 2,3-dioxygenase-1 is the first and rate-limiting enzyme responsible for converting TRP to KYN outside of the liver. We found that IDO1 levels increased after CMS, which suggested IDO1 is an immune mediator in MDD pathogenesis (Murakami and Saito, 2013; Wang et al., 2018). IDO1 activity can be directly inhibited by reactive oxygen species (Marin et al., 2017) or activated by several pro-inflammatory cytokines, including IFNs (IFN-α, -β, and -γ), TNF-α, and IL-6 (Galley et al., 2014). High IDO1 activity may disrupt neurotransmitter balance, which further affects behavior and mood. Similar to previous studies, we found that IDO1 activity in CMS was elevated (Martin-Hernandez et al., 2018), indicating that TRP was more likely to be degraded through the KYN pathway and away from serotonin biosynthesis. IDO1 expression appeared to be dependent on *Lactobacillus* levels, as members of the *Lactobacillus* family can produce high levels of reactive oxygen species (Marin et al., 2017). Reactive oxygen species inhibit IDO1, leading to changes in neurotransmitter levels in the brains. This may be one of the mechanisms through which *Lactobacillus* modulates behavior. Our data suggested that the mixture of these three probiotic strains reduces IDO1 expression, and it has been reported in previous studies that several *Lactobacillus* strains inhibit IDO1 expression (Lau et al., 2011; Vujkovic-Cvijin et al., 2015; Marin et al., 2017).

Inflammation plays a key role in the pathogenesis of depression (Pfau et al., 2018). From our results, the beneficial immunoregulatory effects of fluoxetine and probiotics were another mechanism of neurobehavioral regulation. Disrupting the gut microbiota by CMS may contribute to exaggerated inflammation and immune dysregulation, and the two main benefits of probiotics are supporting healthy digestive and immune systems. Recent studies have shown that *Lactobacillus* affects a multitude of aspects of human physiology. Many bacteria in the genus *Lactobacillus* can be used as probiotics and are distinguished for their immunomodulatory actions and therapeutic applications. Micro integral membrane protein, a newly discovered anti-inflammatory protein of *L. plantarum*, protects gut barrier function and modulates microbiota and inflammatory cytokines (Yin et al., 2018). *Lactobacillus* are able to control a wide range of bacterial strains by secreting antimicrobial factors (Mazaya et al., 2015). In our study, the probiotics-treated group showed reduced TNF-α and IFN-γ levels following CMS, which agreed with previously published studies that described the anti-inflammatory activity of *Lactobacillus* and *Bifidobacterium* (Consonni et al., 2018). Cytokines and immune cells are closely related to blood-brain barrier integrity, as they can enhance blood-brain barrier permeability by inducing injury and leakage of the blood-brain barrier endothelium. This can result in the onset of depression or exacerbate depression symptoms (Lichtblau et al., 2013). The effects of *Lactobacillus* and *Bifidobacterium* may be attributed to distinct changes in immune homeostasis and significant adaptations in immune responses (Consonni et al., 2018).

This study has a few limitations: First, we ignore the effects of behavioral testing on the gut microbiota-inflammation-brain axis. A battery of behavioral tests in mice is also a potent psychophysiological stressor that may alter endocrine, neurochemical, immune function, and microbiota, but very little research has been done in this field (Pawlak et al., 2005). Previous studies have found that serum corticosterone concentration was significantly increased after 15 min post FST exposure, but by 120 min after exposure it returned to the pre-test level (Connor et al., 1998). Since the stress impact depends on the intensity and duration, we applied the following strategy to avoid the effects of behavioral tests on animals, such as: only one behavioral test per day; used 6 min FST duration rather than the traditional 15 min; food deprivation was not applied for SPT; sample collection on the second day after completion of behavioral tests. In addition, during the behavioral testing, we minimized the impact of environmental factors such as factors of noise, light, temperature, handling, in-house transport. Nevertheless, baseline characterization of gut microbiota, inflammation and IDOl activity in the hippocampus of naive mice is still needed. Second, the probiotic-mediated effect exhibits a strain-specific properties. We evaluated the anti-depressive effect of a mixture of three strains, therefore it is hard to make clear which strain make sense or synergistic effect. Further work is needed to evaluate anti-depressive effect of a single strain and the impact on the gut microbiota-inflammation-brain axis.

## Conclusion

Taken together, our results demonstrated that the intestinal microbiome structure and community, and proinflammatory cytokines and IDO1 protein levels in the hippocampus were robustly altered in mice that underwent CMS, demonstrating the inextricable relationship between the gut microbiota, and the immune and nervous systems. Moreover, our data suggested that probiotics can modulate stress-related behaviors in rodents by decreasing hippocampal levels of proinflammatory cytokines (IFN-γ and TNF-α), and direct or inflammatory-mediated inhibition of IDO1 activity. This work supported microbe-based interventions for stress-related disorders, and thus, future studies are required to reveal the exact mechanisms of how the gut microbiota-inflammation-brain axis affects depressive phenotypes. Moreover, assessing multiple neural pathways will be required to determine which are involved in the probiotics-induced attenuation of stress-related behaviors. Through these studies, we expect to develop commensals and more particularly defined member of the microbiota with anti-depressive responses.

## Author Contributions

NL and XL conceived and designed the experiments. NL, YW, and QW performed the experiments and analyzed the data. NL and YL performed the behavioral test. NL, AS, and YJ performed the biochemical analysis. NL wrote the paper.

## Conflict of Interest Statement

The authors declare that the research was conducted in the absence of any commercial or financial relationships that could be construed as a potential conflict of interest.

## Abbreviations

CMS: chronic mild stress
IDO1: indoleamine 2,3-dioxygenase-1
IFN: interferon
IL: interleukin
KYN: kynurenine
MDD: major depressive disorder
PCoA: principal coordinate analysis
TNF-α: tumor necrosis factor-α
TRP: tryptophan
